# Phylogeny and biogeography of the remarkable genus *Bondarzewia* (Basidiomycota, Russulales)

**DOI:** 10.1038/srep34568

**Published:** 2016-09-29

**Authors:** Jie Song, Jia-Jia Chen, Min Wang, Yuan-Yuan Chen, Bao-Kai Cui

**Affiliations:** 1 Institute of Microbiology, Beijing Forestry University, P.O. Box 61, Beijing 100083, China

## Abstract

*Bondarzewia* is a conspicuous and widely distributed mushroom genus, but little is known about its origin and biogeography. Here, we investigated the systematics and biogeography of *Bondarzewia* species using multi-locus phylogenetic analysis. Four genetic markers, including the internal transcribed spacer (ITS), large nuclear ribosomal RNA subunit (nLSU), elongation factor 1-α (tef1) and mitochondrial small subunit rDNA (mtSSU), were used to infer the phylogenetic relationships of *Bondarzewia*. We performed Bayesian evolutionary analysis on the gene datasets of the largest and second largest subunits of RNA polymerase II (RPB1 and RPB2). From the results, we inferred that the maximum crown age of *Bondarzewia* is approximately 25.5 million-years-ago (Mya) and that tropical East Asia is likely to be its ancestral area, with three possible expansions leading to its distribution in North America, Europe and Oceania.

*Bondarzewia* Singer (Bondarzewiaceae, Russulales) is a globally distributed genus of mushroom forming fungi. Some species are edible and have medicinal potential^1^,^2^, whereas some are considered to be forest pathogens^3^. *Bondarzewia* can be mistaken for the mycorrhizal genus *Lactarius*^4^. Phylogenetically, *Bondarzewia* forms sister relationship with the genus *Heterobasidion* in Bondarzewiaceae, but *Lactarius* is closed to *Russula* in Russulaceae^5^. Species of *Bondarzewia* are not mycorrhizal^5^, and eleven species are currently accepted in the genus: *B. dickinsii* (Berk.) Jia J. Chen, B.K. Cui & Y.C. Dai, *B. podocarpi* Y.C. Dai & B.K. Cui, *B. submesenterica* Jia J. Chen, B.K. Cui & Y.C. Dai and *B. tibetica* B.K. Cui, J. Song & Jia J. Chen (reported from Asia)^6^,^7^,^8^, *B. mesenterica* (Schaeff.) Kreisel (from Europe)^9^, *B. berkeleyi* (Fr.) Bondartsev & Singer and *B. occidentalis* Jia J. Chen, B.K. Cui & Y.C. Dai (from North America)^7^,^10^, *B. kirkii* J.A. Cooper, Jia J. Chen & B.K. Cui, *B. propria* (Lloyd) J.A. Cooper and *B. retipora* (Cooke) M.D. Barrett (from Oceania), and *B. guaitecasensis* (Henn.) J.E. Wright from (South America)^7^,^11^.

Previous studies of *Bondarzewia* were mainly based on morphological characters, and three species from North and South America were confirmed^10^,^11^,^12^. Recent, phylogenetic analyses of *Bondarzewia* have been based on the sequences of the internal transcribed spacer (ITS) and the large nuclear ribosomal RNA subunit (nLSU)^6^,^7^,^8^. Five new species and three new combinations were established with clear interspecific affinities: *B. dickinsii* and *B. occidentalis* group together and show a close relationship; *B. podocarpi*, *B. propria*, *B. kirkii* and *B. retipora* cluste together and from two sister groups with different hosts; *B. submesenterica and B. tibetica* are closely related with *B. mesenterica*.

*Bondarzewia* is a marcofungal genus with a few species and comparatively complete records worldwide, and the interspecific affinities are clear. However, a comprehensive estimation of divergence time is lacking, and the biogeography of the *Bondarzewia* mushrooms is not well understood. Molecular phylogeny has been widely used to delineate the lineages and their biogeographic distribution, and major ecological and geological events can be dated more accurately by applying the molecular clock, with the proper calibrations determined by fossils to gene phylogenies^13^. Fungal fossil records are rare and not frequent in the evolutionary history of fungi. Recent molecular studies on basidiomycetous fungi determined the divergence between Basidiomycota and Ascomycota (i.e., 582 Mya) based on a 400-million-year-old fossil of *Paleopyrenomycites devonicus*^13^,^14^,^15^,^16^.

The aims of the present study were to assess the divergence and biogeography of *Bondarzewia*. Therefore, we carried out multi-locus phylogenetic analyses with five ribosomal DNA genes and one mitochondrial gene, *i.e*., ITS, nLSU, elongation factor 1-α (tef1), the largest and second largest subunits of RNA polymerase II (RPB1 and RPB2, respectively), and mitochondrial small subunit rDNA (mtSSU). These genes are common in fungi identification which could delimitate the species very well.

## Results

### Phylogenetic analysis with the combined dataset

The ITS dataset included sequences from 27 fungal samples representing 13 taxa. The dataset had an aligned length of 576 characters, of which 418 are constant, 17 are variable and parsimony uninformative, and 141 are parsimony informative. The nLSU dataset included sequences from 26 fungal samples representing 12 taxa. The dataset had an aligned length of 881 characters, of which 819 are constant, 2 are variable and parsimony uninformative, and 60 are parsimony informative. The mtSSU dataset included sequences from 22 fungal samples representing 11 taxa. The dataset had an aligned length of 485 characters, of which 389 are constant, 45 are variable and parsimony uninformative, and 53 are parsimony informative. The tef1 dataset included sequences from 23 fungal samples representing 11 taxa. The dataset had an aligned length of 540 characters, of which 389 are constant, 8 are variable and parsimony uninformative, and 143 are parsimony informative. The best fitting model identified for the single gene datasets (ITS, nLSU, mtSSU and tef1) were the same: General Time Reversible + Proportion Invariant + Gamma (GTR + I + G). Maximum parsimony (MP), Maximum likehood (ML), and Bayesian inference (BI) analyses yielded similar tree topologies for each gene, and only only the support values in the nodes are different. Only the ML tree with maximum parsimony (MP), maximum likelihood (BS) and Bayesian posterior probabilities (BPP) value were provided (see Supplementary Fig. S1). The phylogenetic results based on mtSSU did not clearly differentiate taxa of *Bondarzewia*, and revealed weakly supported clades or subclades (Fig. S1). ITS, nLSU or tef1 dataset could clearly differentiate the taxa of *Bondarzewia* (Fig. S1).

**Figure 1:**
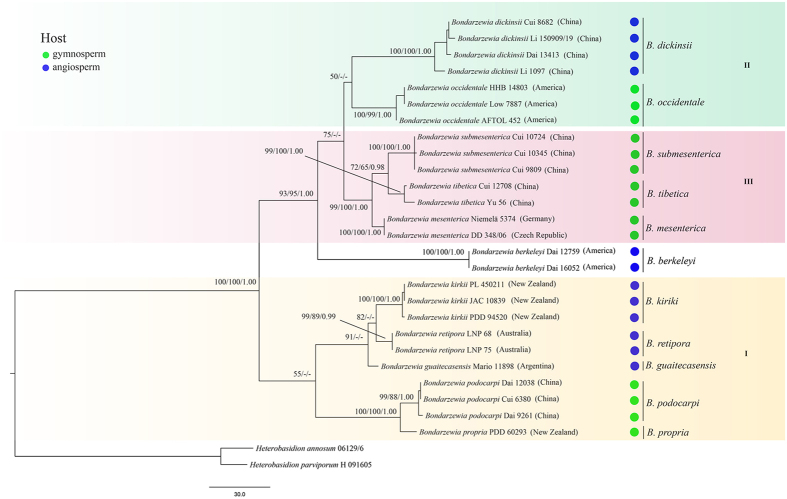
Phylogenetic tree within *Bondarzewia* inferred from the Maximum Parsimony (MP) analysis based on the ITS + nLSU + mtSSU + tef1 dataset. Branches are labeled for MP/BS and BPP values greater than 50% and 0.95, respectively.

The ITS + nLSU + mtSSU + tef1 sequence matrix contained 28 taxa and 2483 aligned base pairs (bp), of which 576 bp, 881 bp, 485 bp and 540 bp from ITS, nLSU, mtSSU and tef1, respectively. The best fitting model identified for the combined dataset were (GTR + I + G). The MP analysis yielded 4 equally parsimonious trees (TL = 661, CI = 0.834, RI = 0.917, RC = 0.764, HI = 0.166), and only the position of species in each clades behave little different. ML analysis and BI yielded similar tree topologies to the one inferred by MP analysis and only the support values in the nodes are different (Fig. 1). The taxa of *Bondarzewia* could differentiate clearly and the backbones of the phylogenetic tree were highly supported.

Based on the combined dataset analyses, our results showed that the genus *Bondarzewia* forms a group with strongly support (100% MP, 100% BS, 1.00 BPP) and can be divided into three distinctive clades along with *B. berkeleyi* (Fig. 1). Clade I is moderately supported by MP analyses (55% MP). This clade comprises five species from East Asia, Oceania and South America. Clade II is composed of two species and is moderately supported by MP analyses (50% MP). Within this clade, *B. dickinsii* is distributed in East Asia and *B. occidentale* covers the East Coast of America. Clade III is composed of three species from Eurasia and is well-supported (99% MP, 100% BS, 1.00 BPP).

### Divergence of *Bondarzewia* lineages

**Figure 2:**
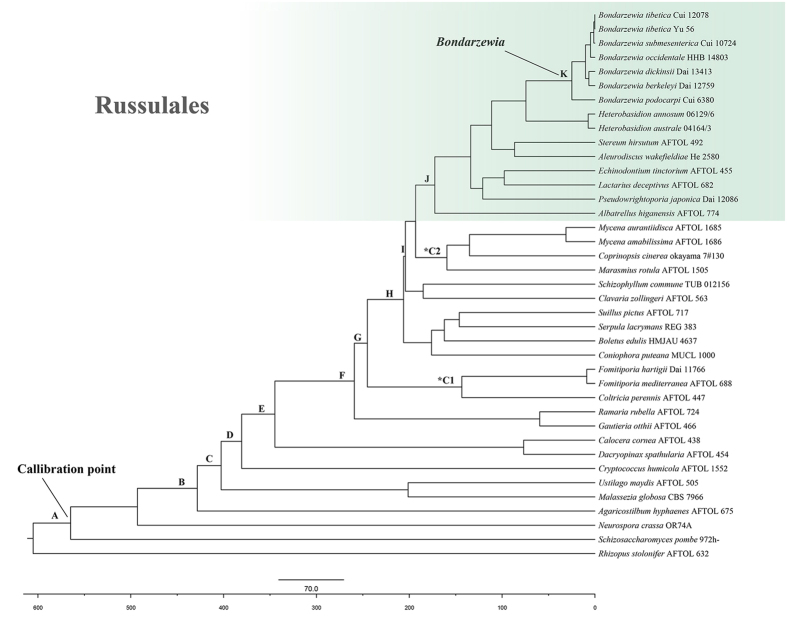
Chronogram and estimated divergence times of *Bondarzewia* generated by molecular clock analysis using the RPB1 and RPB2 dataset. The chronogram was obtained using the Ascomycota–Basidiomycota divergence time of 582 Mya as the calibration point. The calibration point and objects of this study are marked in the chronogram. The geological time scale is in millions of years ago (Mya).

The alignment of the two datasets (RPB1 and RPB2), which are 1554 and 1462 bp in length respectively, consisted of 39 taxa. Analyses calibrated by *P. devonicus* (Fig. 2), 582 Mya between Ascomycota and Basidiomycota, estimate the divergence time of Russulales at 173.78 ± 0.47 Mya (127.42–220.43 Mya, 95% HPD) that meet the constraint of Russulales. The initial diversification of *Bondarzewia* is at Late-Oligocene, 25.54 ± 0.17 Mya (15.65–37.18 Mya, 95% HPD). The estimated divergence time of two fossil records point for comparison, *Archaeomarasmius leggetti* Hibbett, D. Grimaldi & Donoghue and *Quatsinoporites cranhamii* S.Y. Sm., Currah & Stockey, are 159.45 ± 0.45 Mya (118.75–208.64 Mya, 95% HPD) and 143.34 ± 0.42 Mya (93.13–195.41 Mya, 95% HPD). The estimated divergence times for other nodes are summarized in Table 1.

### Historical biogeography of *Bondarzewia*

**Figure 3:**
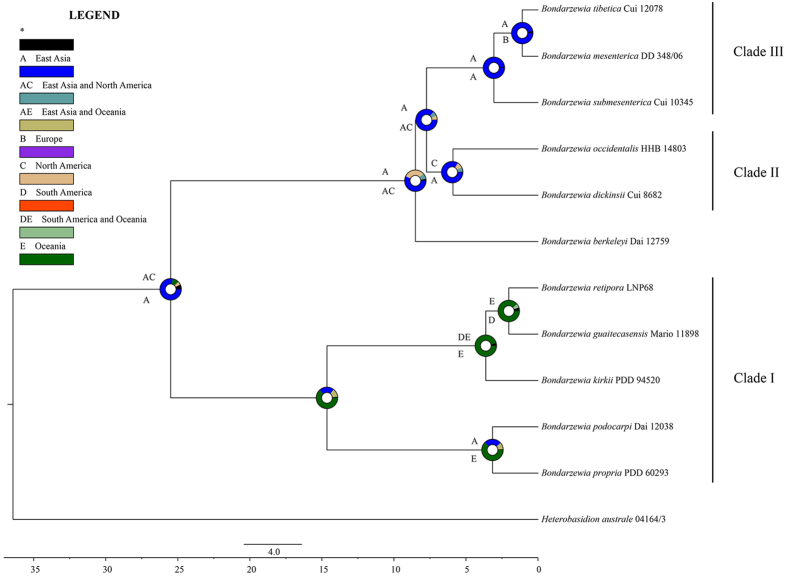
Divergence time estimation and ancestral area reconstruction of *Bondarzewia* using the ITS dataset. The chronogram was obtained by molecular clock analysis using BEAST. The pie chart in each node indicates the possible ancestral distributions inferred from Bayesian Binary MCMC analysis implemented in RASP. The characters above and below each branch identify the possible ancestral distribution estimated by maximum likelihood-based program LAGRANGE. The color key lists the possible ancestral ranges at different nodes; black with an asterisk represents other ancestral ranges.

**Figure 4:**
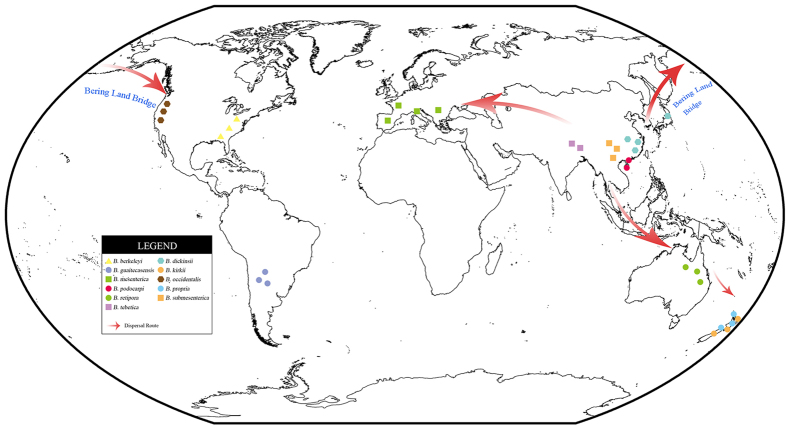
Map of the geographic distribution of *Bondarzewia* and its possible dispersal routes generated by ArcGIS v10.1 (http://esri.com/arcgis). A hypothetical schematic depicting of the place of origin, their migration routes, and the rapid radiation and speciation of *Bondarzewia*.

The inferred historical biogeographic scenarios from the analyses conducted using LAGRANGE^17^ and RASP^18^ are shown in Fig. 3. Our biogeographical analyses indicated that, of the 11 phylogenetic species that were identified in this lineage, 4 (37%), 3 (27%), 2 (18%), 1 (9%), and 1 (9%) of the species were reported to be from East Asia, Oceania, North America, Europe and South America, respectively. The Bayesian binary Markov chain Monte Carlo analysis showed the East Asia presented the highest probability (78%) of being the ancestral area of *Bondarzewia*. The maximum likelihood-based estimation also provided strong support for East Asia being the ancestral area. In addition, the basal species (*B. guaitecasensis*, *B. kirkii*, *B. podocarpi*, *B. propria*, and *B. retipora*) exhibited a pantropical distribution pattern (Figs 1 and 3). These findings support that *Bondarzewia* originated in tropical East Asia. Meanwhile, the East Asian and North American ancestral origins of the Holarctic *Bondarzewia* species are also supported by LAGRANGE. The three clades indicated three kinds of intercontinental distribution patterns respectively: East Asia–Oceania–South America, East Asia–North America, and East Asia–Europe (Fig. 4).

## Discussion

In this study, we presented results from investigations on the phylogeny, origin and biogeography of the genus *Bondarzewia*. Three major clades and one isolate species comprising 11 species were identified within the genus (Fig. 1). In the following discussion, we focused on the features of the major clades and their distribution patterns.

Clade I is weakly supported and includes five taxa representing two sister groups (Fig. 1). *B. podocarpi* and *B. propria* formed a significantly supported gymnosperm-associated group (100% MP, 100% BS, 1.00 BPP); they share dimitic hyphal structure, have similarly sized pores (2 per mm) and cyanophilous basidiospores. To date, *B. podocarpi* has been found only in southern tropical China on *Podocarpus* and *Dacrydium*, which are native flora of the Southern Hemisphere^6^,^7^. *B. propria* was first found in New Zealand and was associated with the endemic plants *Dacrydium cupressinum* and *Agathis australis*^7^. *B. retipora*, *B. kirkii* and *B. guaitecasensis* formed an angiosperm-associated species group (91% MP). These three species grow on native evergreen angiosperm trees of the Nothofagaceae, Meliaceae or Lamiaceae families, and morphologically, they share orange pileal surfaces and a dimitic hyphal structure^7^,^11^. The data from the current work indicated close close relationships among these species.

*B. dickinsii* and *B. occidentale* were grouped together with low support (50% MP) in our phylogeny. They both grow in temperate areas and have white pore surfaces and cyanophilous basidiospores^7^. *B. dickinsii* is associated with Fagaceae such as *Quercus* and *Castanea*, and has been found from East Asia; however, *B. occidentale* grows on gymnosperm trees such as *Picea* and *Tsuga*, and has been found only from West America so far^7^.

The East Asian species *B. submesenterica* and *B. tibetica* and the European species *B. mesenterica* were grouped together (Fig. 1). They all produce brown pileal surfaces, cream pore surfaces, and grow mainly on conifers. *B. submesenterica* and *B. tibetica* were found in temperate areas of the Hengduan-Himalayan region, which is a global biodiversity hotspot located in China. *B. mesenterica* is common in Central-East and South Europe, which was also a refuge for organisms^19^. The host plants such as *Abies*, *Picea* and *Pinus* also have this kind of distribution pattern^20^,^21^. These results indicated that the appropriate growth environments of refuges may have guaranteed the survival of *Bondarzewia* during the Quaternary Ice Age.

*Bondarzewia berkeleyi* fromed a lineage in the multi-loci phylogeny (Fig. 1). This species has been delimited as an East American species and grows mainly on deciduous plants such as *Quercus* and *Acer*^7^,^10^. Phylogenetically, *B. berkeleyi* diverged the earliest of the Holarctic *Bondarzewia* species and occupies a basal and separate position.

The maximum crown age of *Bondarzewia* was estimated to be around the Late Oligocene (25.54 ± 0.17 Mya). During that period, the general landforms of the modern world had already formed, and dramatic climate changes was ongoing^22^,^23^. The resultant polar ice sheets advanced and retreated several times and covered most of North America and northern Europe until the Last Glacial Maximum (ca. 18,000 years ago)^23^. Meanwhile, belts of relatively arid climates in the centers of Eurasia and North America formed^24^. Previous study on organisms, including the host plants of *Bondarzewia* such as *Abies*, *Salix* and *Populus*, proved that dispersal, extinction and speciation occurred during this time because of the dramatic climate changes^20^,^25^,^26^,^27^,^28^,^29^.

Our results supported that *Bondarzewia* originated in tropical East Asia. However, the basal species were associated with Nothofagaceae, Meliaceae, Lamiaceae, Podocarpaceae and Araucariaceae; according to the co-evolution of fungi and host plants, the Gondwana origin cannot be rejected because Araucariaceae, Podocarpaceae, Nothofagaceae and Meliaceae are thought to have originated in Gondwana^30^,^31^,^32^,^33^,^34^. The Holarctic species including Clade II, Clade III and *B. berkeleyi* diverged later (8.5 Mya) and were restricted to temperate zones and temperate plants such as *Picea*, *Abies*, *Quercus* and *Castanea*^7^,^8^. The temperate habit and late divergence time suggest that an adaptation to temperate climates occurred.

Species in the Clade I appeared the earliest in our phylogenies and were distributed in South China, New Zealand, Australia and South America (Figs 1 and 3). Species could have migrated within tropical South East Aisa, New Zealand and Australia (Fig. 4), when the Oceania and Asian plates were connected after their collision close to the Oligo-Miocene boundary^35^,^36^. The evolutionary studies of organism on either side of Wallace’s Line could clearly confirm the migration between Oceania and Asian plates^37^. Interestingly, our data suggest that *B. guaitecasensis*, *B. kirkii* and *B. retipora* are closely related and diverged comparatively recently (3.6 Mya). As we all known, there are twenty thousand islands in South Pacific such as Solomon island archipelago, New Caledonia and Hawaii islands. The scaly tree ferns has been proved to dispersal between Oceania and South America by wind via the lightweight spores^38^. We speculate that the wind and ocean current drive the dispersal of *Bondarzewia* in South Pacific island by island.

Two sister species within Clade II, *B. dickinsii* and *B. occidentale*, exhibited an East Asia–Western North America temperate disjunct distribution (Figs 1 and 3), and their divergence time was estimated at approximately 5.9 Mya, implying that dispersals between East Asia and Western North American occurred, probably via the Bering Land Bridge (BLB) prior to their divergence (Fig. 4). The BLB separated from 5.5–5.4 Mya^39^,^40^, and connected the East Asia and North America before that time. The BLB has been confirmed as a route in the dispersal event of many other organisms^14^,^15^,^41^,^42^,^43^. The divergence of *B. dickinsii* and *B. occidentale* may result from the separate of BLB and the extinction of species occurred. At the same time, the continuous significant climate changes between 15 Mya and the Last Glacial Maximum may have played important role in breaking the biotic connections between Tertiary floras of East Asia and North America and in facilitating allopatric speciation^22^,^23^,^44^.

An East Asian–European allopatric speciation was also inferred for *B. submesenterica*, *B. tibetica* and *B. mesenterica* (Figs 3 and 4). The estimated divergence occurred at approximately 3.1 Mya, and East Asia was inferred to be the most likely ancestral area. Long-distance dispersal between Europe and East Asia may have occurred, which was common in the immigration of plants such as *Quercus*, *Salix*, *Populus*, *Picea*, *Abies* and *Larix*^20^,^21^,^25^,^28^,^29^,^45^. We speculate that the severe climate changes that occurred since 15 Mya and the sebsequent aridification in Central Eurasia^22^ may have been the causes of the divergence.

The East American species, *B. berkeleyi*, occupies the basal and separate position in the phylogenetic tree of the Holarctic *Bondarzewia* species (Figs 1 and 3). The dating analysis inferred an earlier divergence time (8.5 Mya). Its hosts such as *Quercus* and *Acer* have an extensive distribution in the Northern Hemisphere. We speculate its ambiguous affinities with other species may relate to an incomplete sampling and undescribed species in Central American. Alternatively, *B. berkeleyi* could be an earlier relic of *Bondarzewia* in the Western North America and the extinction of this species in the Holarctic region occurred after it originated from tropical East Asia. The separate position of East American B. berkeleyi deserves a detailed study.

## Conclusion

The monophyletic genus *Bondarzewia* originated in the tropical zone of East Asia and has diverged since 25.54 ± 0.17 Mya (15.65–37.18 Mya, 95% HPD). The severe climate changes and the resulting reduction in sea-levels and aridification of the center of continents shaped the evolutionary history of *Bondarzewia* via the co-diversification of the fungi and their host plants. Three intercontinential distribution patterns were recognized: East Asia–Oceania–South America, East Asia–North America, and East Asia–Europe. More samples and sequences are needed to improve our understanding of the biodiversity and biogeography of *Bondarzewia*. The genus diversity still deserves a further study because the samples are incomplete in many places such as Central Asia, Siberia, Indonesia and South Africa, and the evolution history of some species are still ambiguous.

## Materials and Methods

### Taxa sampling

The sampled taxa, their genetic markers, and their GenBank accession numbers are provided (Table 2). Specimens from East Asia (EA), Europe (EUR), North America (NA), Oceania (OC), and South America (SA) were studied for their morphological characters. The outgroup taxa were determined based on the previous phylogenetic study that focused on the diversity of *Bondarzewia*^8^.

### DNA extraction, PCR, and DNA sequencing

A rapid plant genome DNA extraction kit (Aidlab Biotechnologies Co., Ltd, Beijing, China) and the primers listed in Table 3 were used to obtain PCR products from dried specimens and cultures according to the manufacturer’s instructions with modifications. The PCR protocols for ITS, nLSU, mtSSU, tef1, RPB1 and RPB2 have been described previously publication by the same research group^46^. The PCR products were purified and sequenced at the Beijing Genomics Institute (China) using the same primers. All newly generated sequences were deposited in GenBank (http://www.ncbi.nlm.nih.gov/).

### Sequence alignments and phylogenetic analyses

The sequences of *Heterobasidion annosum* (Fr.) Bref. and *Heterobasidion parviporum* Niemelä & Korhonen were used as outgroups^8^. Phylogenetic analyses was applied to single-locus genealogies for ITS, nLSU, mtSSU and tef1, and concatenated dataset that contained the ITS + nrLSU + mtSSU + tef1 sequences. Initially, the four genes were aligned using MAFFT 6 (http://mafft.cbrc.jp/alignment/server/)^47^ with “G-INS-I” strategy and then the alignment was manually optimized in BioEdit^48^. Finally, the four gene fragments were concatenated with SEAVIEW 4^49^ for further phylogenetic analysis. One thousand partition homogeneity test (PHT) replicates of ITS, nrLSU, mtSSU, and tef1 sequences were tested by PAUP* version 4.0b10 (Swofford, 2002) to determine whether the partitions were homogeneous. The PHT results indicated all the DNA sequences display a congruent phylogenetic signal (P value = 0.02).

ML, MP and BI methods were used to analyze the compiled datasets. A suitable substitution model for each partition of the dataset was determined using the Akaike Information Criterion implemented in MrMODELTEST2.3^50^. PAUP* 4.0b10^51^ was used for MP analysis. All characters were equally weighted, and gaps were treated as missing data. Trees were inferred using the heuristic search option with TBR branch swapping and 1000 random sequence additions. Max-trees was set to 5000, branches of zero length were collapsed, and all parsimonious trees were saved. Clade robustness was assessed using bootstrap analysis with 1000 replicates. Descriptive tree statistics including the tree length (TL), consistency index (CI), retention index (RI), rescaled consistency index (RCI), and homoplasy index (HI), were calculated for each maximum parsimony tree generated.

ML searches conducted with RAxML-HPC2^52^ on Abe through the Cipres Science Gateway (www.phylo.org) involved 100 ML searches under the GTR + GAMMA model; all model parameters were estimated by the program. Only the best maximum likelihood tree from all searches was kept. In addition 100 rapid bootstrap replicates were run with the GTR + CAT model to assess the reliability of the nodes.

BI was examined with MrBayes3.1.2^53^ with a general time-reversible model of DNA substitution and an inverse-gamma distribution rate variation across sites. Four Markov chains were run from the random starting tree for 1 million generations for the combined datasets. Trees were sampled every 100 generations. The burn-in was set to discard the first 25% of the trees. A majority rule consensus tree of all the remaining trees was used to calculate BPP.

Branches that received bootstrap support for MP, BS and BPP greater than or equal to 75% (MP/BS) and 0.95 (BPP) were considered to be significantly supported.

### Divergence time estimation

Several studies have attempted to date the evolutionary splits of fungi using various calibration strategies^14^,^15^,^41^. Here, we used internal calibration to determine the divergence time between Ascomycota and Basidiomycota, 582 Mya, with the 400-million-years-old fossil *Paleopyrenomycites devonicus* Taylor, Hass, Kerp, M. Krings & Hanlin^16^. A normal distribution was applied by setting the mean and the standard deviation to 582.5 and 50.15, respectively^16^. One constraint was applied: the initial diversification of the Russulales was set at 189 Mya, consistent with the conservative estimated divergence time for the plant family Pinaceae^54^. Co-divergence between fungal lineages and their plant hosts suggest that Russulales and its allies occurred around, or slightly later than, the time of the diversification of Pinaceae^21^,^55^.

The BEAST 1.8.0 software package was used to estimate divergence times^56^. The two gene fragment, RPB1 and RPB2, were concatenated for molecular dating. We retrieved the sequences of six additional species—*Marasmius rotula* (Scop.) Fr., *Mycena amabilissima* Peck, *M. aurantiidisca* (Murrill) Murrill, *Fomitiporia hartigii* (Allesch. & Schnabl) Fiasson & Niemelä, *F. mediterranea* M. Fisch., and *Coltricia perennis* (L.) Murrill—as representative taxa of the initial diversification of mushroom-forming fungi (based on the 90-million-year-old fossil, *A. leggetti* Hibbett, D. Grimaldi & Donoghue)^57^ and the divergence of the Hymenochaetaceae (based on the 125-million-year-old fossil, *Q. cranhamii* S.Y. Sm., Currah & Stockey^58^).

First, we used BEAUti to generate xml files that were executable in BEAST. The RPB1 and RPB2 datasets were set as two partitions, the substitution and molecular clock models were set as unlinked, and the inferred trees were set as linked. For both partitions, the GTR model was chosen as the best substitution model by MrModelTest, and a relaxed lognormal model was employed for molecular clock analysis^59^. The tree prior was set to Yule speciation. For each analysis, two independent runs were conducted for 100 million generations. Log files of the two runs were combined using LogCombiner by setting the first 10% of the logs as burn-ins and then analyzed in Tracer 1.5 (http://tree.bio.ed.ac.uk/software/figtree/tracer). The resulting trees were also combined, interpreted in TreeAnnotator, and viewed in FigTree 1.4.0 (http://tree.bio.ed.ac.uk/software/figtree/).

We also estimated the divergence time of the main nodes in *Bondarzewia* using a mini ITS dataset containing representatives of all 11 species. The estimated crown age of the genus *Bondarzewia* inferred by the RPB1 and RPB2 data was used to calibrate the ITS phylogeny by setting the prior to a normal distribution. The other procedures were the same as the ones applied in the estimation using the RPB1 and RPB2 datasets. The most recent common ancestors were only defined for the major clades that were well-supported in the ITS + nLSU + mtSSU + TEA phylogenies.

### Inferring the geographic center of of *Bondarzewia*’s origin

The phylogeny and divergence inferred from the ITS dataset were used to reconstruct the possible historical distributions of *Bondarzewia* lineages. Both the maximum likelihood-based estimations implemented in LAGRANGE and the Bayesian binary Markov chain Monte Carlo analysis provided by RASP v3.2 were used. The geographic distributions of *Bondarzewia* lineages were classified into five areas: East Asia, Europe, North America, South America and Oceania. The probabilities of dispersal were estimated according to the divergence times inferred earlier in this study between different areas as previously summarized^60^. Bayesian binary analysis was conducted in RASP by setting the generations to 10 million and by discarding the first 10% of samples as burn-ins; the other parameters used were the default settings. ArcGIS v10.1 (http://esri.com/arcgis) was used to visualize the geographic distribution and possible dispersal routes of *Bondarzewia*.

## Additional Information

**How to cite this article**: Song, J. *et al*. Phylogeny and biogeography of the remarkable genus *Bondarzewia* (Basidiomycota, Russulales). *Sci. Rep*. **6**, 34568; doi: 10.1038/srep34568 (2016).

## Supplementary Material

Supplementary Information

## Figures and Tables

**Table 1:** Estimated divergence times of each time.

Node	Mean ± standard error	95% HPD	Node	Mean ± standard error	95% HPD
A: Ascomycota/Basidiomycota	564.76 ± 0.34	467.45–666.26	H: Boletales/Agaricomycetes	207.67 ± 0.60	154.78–260.51
B: Ustilaginomycotina/Basidiomycota	429.61 ± 0.53	335.21–529.36	I: Agaricales/Russulales	205.64 ± 0.59	153.73–258.69
C: Pucciniomycotina/Agaricomycotina	404.45 ± 0.53	312.45–497.78	J: Russulales	173.78 ± 0.47	127.42–220.43
D: Tremellomycetes/Agaricomycotina	382.31 ± 0.53	293.36–473.40	K: Bondarzewia	25.54 ± 0.17	15.65–37.18
E: Dacrymycetes/Agaricomycetes	346.66 ± 0.53	261.10–430.92	*C1: Hymenochaetaceae	143.34 ± 0.42	93.13–195.41
F: Gomphales/Agaricomycetes	261.17 ± 0.58	196.72–329.66	*C2: mushroom-forming fungi	159.45 ± 0.45	118.75–208.64
G: Hymenochaetales/Agaricomycetes	247.18 ± 0.57	184.07–310.49			

Fossil record (for comparison): *C1: *Quatsinoporites cranhamii*, 125–130 Mya ; *C2: *Archaeomarasmius leggetti*, 90 Mya (minimum age).

**Table 2:** Information of sequences used in this study.

Species	Sample no.	GenBank accessions					
		ITS	nLSU	RPB1	RPB2	tef1	mtSSU
*Albatrellus higanensis*	AFTOL-ID 774	—	—	AY788846	AY780935	—	—
*Aleurodiscus wakefieldiae*	He 2580	—	—	KX577720^a^	KX577723^a^	—	—
*Agaricostilbum hyphaenes*	AFTOL-ID 675	—	—	AY788845	AY780933	—	—
*Boletus edulis*	HMJAU 4637	—	—	KF112586	KF112704	—	—
*Bondarzewia berkeleyi*	Dai 12759	KJ583202	KJ583216	KX066152^a^	KX066162^a^	KX066138^a^	KX066169^a^
*B. berkeleyi*	Dai 16052	KX263720^a^	KX263722^a^	—	—	—	—
*B. dickinsii*	Cui 8682	KJ583209	KJ583223	KX066150^a^	KX066160^a^	KX066136^a^	KX066167^a^
*B. dickinsii*	Dai 13413	KJ583210	KJ583224	KX066151^a^	KX066161^a^	KX066137^a^	KX066168^a^
*B. dickinsii*	Li 150909/19	KX263721^a^	KX263723^a^	—	—	—	—
*B. dickinsii*	Li 1097	FJ644288	—	—	—	—	—
*B. guaitecasensis*	Rajchenberg 11898	FJ644287	—	—	—	—	KX066175^a^
*B. kirkii*	PDD 94520	KJ583215	KJ583229	—	—	KX252748	KX066180^a^
*B. kirkii*	JAC 10839	KJ734674	KM067469	—	—	KX252747	KX066179^a^
*B. kirkii*	PL 450211	KJ583214	KJ583228a	—	—	KX252746^a^	KX066178^a^
*B. mesenterica*	DD 348/06	KM243328	KM243331	—	—	KX066147^a^	KX066182^a^
*B. mesenterica*	Niemelä 5374	KM067468	KM067470	—	—	KX066146^a^	KX066181^a^
*B. occidentalis*	HHB 14803	KM243329	KM243332	KX066156^a^	KX066163^a^	KX066142^a^	KX066176^a^
*B. occidentalis*	Lowe 7887	KM243330	KM243333	KX066157^a^	KX066164^a^	KX066143^a^	KX066177^a^
*B. occidentalis*	AFTOL-ID 452	DQ200923	DQ234539	DQ256049	AY218474	DQ059044	
*B. podocarpi*	Cui 6380	KJ583206	KJ583220	KX577718^a^	KX577721^a^	KX252745^a^^a^	KX066174^a^
*B. podocarpi*	Dai 9261	KJ583207	KJ583221			KX252743^a^	KX066172^a^
*B. podocarpi*	Dai 12038	KJ583208	KJ583222			KX252744^a^	KX066173^a^
*B. propria*	PDD 60293	KJ583213	KJ583227				
*B. retipora*	LNP 68	KJ747633	KJ747630			KX066144^a^	
*B. retipora*	LNP 75	KJ747632	KJ747629			KX066145^a^	
*B. submesenterica*	Cui 9809	KJ583203	KJ583217			KX066141^a^	KX066171^a^
*B. submesenterica*	Cui 10345	KJ583204	KJ583218			KX066140^a^	KX066170^a^
*B. submesenterica*	Cui 10724	KJ583205	KJ583219	KJ651627	KJ651720	KX066139^a^	
*B. tibetica*	Cui 12078	KT693202	KT693204	KX066159^a^	KX066166^a^	KX066149^a^^a^	KX066184^a^
*B. tibetica*	Yu 56	KT693203	KT693205	KX066158^a^	KX066165^a^	KX066148^a^	KX066183^a^
*Calocera cornea*	AFTOL-ID 438	—	—	AY857980	AY536286	—	—
*Clavaria zollingeri*	AFTOL-ID 563	—	—	AY857987	AY780940	—	—
*Coltricia perennis*	AFTOL-ID 447	—	—	AY864867	AY218526	—	—
*Coniophora puteana*	MUCL 1000	—	—	GU187451	GU187778	—	—
*Coprinopsis cinerea*	okayama 7#130	—	—	XM_001828525	XM_001829088	—	—
*Cryptococcus humicola*	AFTOL-ID 1552	—	—	DQ645518	DQ645517	—	—
*Dacryopinax spathularia*	AFTOL-ID 454	—	—	AY857981	AY786054	—	—
*Echinodontium tinctorium*	AFTOL-ID 455	—	—	AY864882	AY218482	—	—
*Fomitiporia hartigii*	Dai 11766	—	—	KJ651628	KJ651721	—	—
*F. mediterranea*	AFTOL-ID 688	—	—	AY864869	AY803748	—	—
*Gautieria otthii*	AFTOL-ID 466	—	—	AY864865	AY218486	—	—
*Heterobasidion annosum*	06129/6	KJ583211	KJ583225	KF006499	KF033133	KX252741^a^	KJ651577
*H. australe*	04164/3	—	—	KF033134	KF006500	—	—
*H. parviporum*	H 091605	KJ651503	KJ651561	KJ651657	KJ651750	KX252742^a^	KJ651622
*Lactarius deceptivus*	AFTOL-ID 682	—	—	AY864883	AY803749	—	—
*Malassezia globosa*	CBS 7966			KF706493	KF706518		
*Marasmius rotula*	AFTOL-ID 1505	—	—	DQ447922	DQ474118	—	—
*Mycena amabilissima*	AFTOL-ID 1686	—	—	DQ447926	DQ474121	—	—
*M. aurantiidisca*	AFTOL-ID 1685	—	—	DQ447927	DQ474122	—	—
*Neurospora crassa*	OR74A	—	—	XM959004	AF107789	—	—
*Pseudowrightoporia japonica*	Dai 12086			KX577719^a^	KX577722^a^		
*Ramaria rubella*	AFTOL-ID 724	—	—	AFTOL database	AY786064	—	—
*Rhizopus stolonifer*	AFTOL-ID 632	—	—	AFTOL database	AFTOL database	—	—
*Russula* sp.	H-2009BT109A	—	—	JN389202	JN389208	—	—
*Schizophyllum commune*	TUB 012156			DQ068011	KC904262	—	—
*Schizosaccharomyces pombe*	972h-	—	—	NM001021568	NM001018498		
*Serpula lacrymans*	REG 383	—	—	GU187485	GU187809	—	—
*Stereum hirsutum*	AFTOL-ID 492	—	—	AY864885	AY218520	—	—
*Suillus pictus*	AFTOL-ID 717			AY858965	AY786066		
*Ustilago maydis*	AFTOL-ID 505	—	—	AFTOL database	AY485636	—	—

^a^Newly generated sequences.

**Table 3:** PCR primers used in this study.

Gene*	Primer	Primer sequences (5′-3′)^a^	Reference
ITS	ITS5	GGA AGT AAA AGT CGT AAC AAG G	White *et al*. (1990)
	ITS4	TCC TCC GCT TAT TGA TAT GC	White *et al*. (1990)
nLSU	LR0R	ACC CGC TGA ACT TAA GC	http://www.biology.duke.edu/fungi/mycolab/primers.htm
	LR7	TAC TAC CAC CAA GAT CT	http://www.biology.duke.edu/fungi/mycolab/primers.htm
RPB1	RPB1-Af	GAR TGY CCD GGD CAY TTY GG	Matheny *et al*. (2002)
	RPB1-Cf	CCN GCD ATN TCR TTR TCC ATR TA	Matheny *et al*. (2002)
RPB2	fRPB2-5F	GAY GAY MGW GAT CAY TTY GG	Liu *et al*. (1999); Matheny (2005)
	fRPB2-7cR	CCC ATR GCT TGY TTR CCC AT	Liu *et al*. (1999); Matheny (2005)
mtSSU	MS1	CAG CAG TCA AGA ATA TTA GTC AAT G	White *et al*. (1990)
	MS2	GCG GAT TAT CGA ATT AAA TAA C	White *et al*. (1990)
tef1	983F	GCY CCY GGH CAY CGT GAY TTY AT	http://ocid.NACSE.ORG/research/deephyphae/EF1primer.pdf
	1567R	ACH GTR CCR ATA CCA CCR ATC TT	http://ocid.NACSE.ORG/research/deephyphae/EF1primer.pdf

^a^Degeneracr codes: S = G or C, W = A or T, R = A or G, Y = C or T, N = A or T or C or G, D = G or A or T, M = A or C.

*ITS, internal transcribed spacer region; nLSU, the large nuclear ribosomal RNA subunit; RPB1, the largest subunit of RNA polymerase II; RPB2, the second subunit of RNA polymerase II.

## References

[b1] Boa E. Wild edible fungi: a global overview of their use and importance to people. Non-Wood Forest Product 17, 1–147 (2004).

[b2] Dai Y. C., Yang Z. L., Cui B. K., Yu C. J. & Zhou L. W. Species diversity and utilization of medicinal mushrooms and fungi in China (Review). Int. J. Med. Mushrooms 11, 287–302 (2009).

[b3] Dai Y. C., Cui B. K., Yuan H. S. & Li B. D. Pathogenic wood-decaying fungi in China. Forest Pathol. 37, 105–120 (2007).

[b4] Shernoff L. Easy Edibles: Hen of the Woods. Mushroom: the Journal of Wild Mushrooming 27, 19–20 (2009).

[b5] Larsson E. & Larsson K. H. Phylogenetic relationships of russuloid basidiomycetes with emphasis on aphyllophoralean taxa. Mycologia 95, 1037–1065 (2003). 21149013 10.1080/15572536.2004.11833020

[b6] Dai Y. C., Cui B. K. & Liu X. Y. *Bondarzewia podocarpi*, a new and remarkable polypore from tropical China. Mycologia 102, 881–886 (2010). 20648754 10.3852/09-050

[b7] Chen J. J. . Molecular phylogeny and global diversity of the remarkable genus *Bondarzewia* (Basidiomycota, Russulales). Mycologia 108, 697–708 (2016). 27091389 10.3852/14-216

[b8] Li G. J. . Fungal diversity notes 253–366: taxonomic and phylogenetic contributions to fungal taxa. Fungal Divers. 78, 1–237 (2016).

[b9] Ryvarden L. & Melo I. Poroid fungi of Europe. Syn. Fung. 31, 1–455 (2014).

[b10] Gilbertson R. L. & Ryvarden L. North American polypores 1. Fungiflora, Oslo (1986).

[b11] Singer R. New genera of fungi 12. Hybogaster. Sydowia 17, 12–16 (1964).

[b12] Rajchenberg M. Taxonomic studies on selected Austral polypores. Aus. Sys. Bot. 16, 473–485 (2003).

[b13] Berbee M. L. & Taylor J. W. Dating the molecular clock in fungi – how close are we? Fungal Biol. Rev. 24, 1–16 (2010).

[b14] Cai Q. . Multi-locus phylogeny of lethal amanitas: Implications for species diversity and historical biogeography. BMC Evol. Biol. 14, 143 (2014). 24950598 10.1186/1471-2148-14-143 PMC4094918

[b15] Feng B. . DNA sequences analyses reveal abundant diversity, endemism and evidence for Asian origin of the Porcini. PloS One 7, e37567 (2012). 22629418 10.1371/journal.pone.0037567 PMC3356339

[b16] Taylor T. N., Hass H., Kerp H., Krings M. & Hanlin R. T. Perithecial ascomycetes from the 400 million year old Rhynie chert: an example of ancestral polymorphism. Mycologia 96, 1403–1419 (2004). 16389979

[b17] Ree R. H. & Smith S. A. Maximum likelihood inference of geographic range evolution by dispersal, local extinction, and cladogenesis. Syst. Biol. 57, 4–14 (2008). 18253896 10.1080/10635150701883881

[b18] Yu Y., Harris A. J., Blair C. & He X. J. RASP (Reconstruct Ancestral State in Phylogenies): a tool for historical biogeography. Mol. Phylogenet. Evol. 87, 46–49 (2015). 25819445 10.1016/j.ympev.2015.03.008

[b19] Myers N., Mittermeier R. A., Mittermeier C. G., da Fonseca G. A. B. & Kent J. Biodiversity hotspots for conservation priorities. Nature 403, 853–858 (2000). 10706275 10.1038/35002501

[b20] Aguirre-Planter É. . Phylogeny, diversification rates and species boundaries of Mesoamerican firs (Abies, Pinaceae) in a genus-wide context. Mol. Phylogenet. Evol. 62, 263–274 (2012). 22019929 10.1016/j.ympev.2011.09.021

[b21] Ran J. H., Wei X. X. & Wang X. Q. Molecular phylogeny and biogeography of *Picea* (Pinaceae): Implications for phylogeographical studies using cytoplasmic haplotypes. Mol. Phylogenet. Evol. 41, 405–419 (2006). 16839785 10.1016/j.ympev.2006.05.039

[b22] Tiffney B. H. & Manchester S. R. The use of geological and paleontological evidence in evaluating plant phylogeographic hypotheses in the northern hemisphere tertiary. Int. J. Plant Sci. 162, S3–S17 (2001).

[b23] Zachos J., Pagani M., Sloan L., Thomas E. & Billups K. Trends, Rhythms, and Aberrations in Global Climate 65 Ma to Present. Science 292, 686–693 (2001). 11326091 10.1126/science.1059412

[b24] Milne R. I. Northern hemisphere plant disjunctions: a window on tertiary land bridges and climate change? Ann. Bot. 98, 465–472 (2006). 16845136 10.1093/aob/mcl148 PMC2803576

[b25] Cronk Q. C. B., Needham I. & Rudall P. J. Evolution of Catkins: Inflorescence Morphology of Selected Salicaceae in an Evolutionary and Developmental Context. Front. Plant. Sci. 6, 1030 (2015). 26697024 10.3389/fpls.2015.01030 PMC4671327

[b26] Cortés-Ortiz L. . Molecular systematics and biogeography of the Neotropical monkey genus, Alouatta. Mol. Phylogenet. Evol. 26, 64–81 (2003). 12470939 10.1016/s1055-7903(02)00308-1

[b27] Dick C. W., Abdul-Salim K. & Bermingham E. Molecular Systematic Analysis Reveals Cryptic Tertiary Diversification of a Widespread Tropical Rain Forest Tree. Am. Nat. 162, 691–703 (2003). 14737707 10.1086/379795

[b28] Du S. H. . Multilocus analysis of nucleotide variation and speciation in three closely related Populus (Salicaceae) species. Mol. Ecol. 24, 4994–5005 (2015). 26334549 10.1111/mec.13368

[b29] Manos P. S. & Stanford A. M. The Historical Biogeography of Fagaceae: Tracking the Tertiary History of Temperate and Subtropical Forests of the Northern Hemisphere. Int. J. Plant Sci. 162, S77–S93 (2001).

[b30] Kershaw P. & Wagstaff B. The southern conifer family Araucariaceae: history, status, and value for paleoenvironmental reconstruction. Annu. Rev. Ecol. Syst. 32, 397–414 (2001).

[b31] McLoughlin S. The breakup history of Gondwana and its impact on pre-Cenozoic floristic provincialism. Aust. J. Bot. 49, 271–300 (2001).

[b32] Ornelas J. F., Ruiz-Sanchez E. & Sosa V. Phylogeography of *Podocarpus matudae* (Podocarpaceae): pre-Quaternary relicts in northern Mesoamerican cloud forests. J. Biogeogr. 37, 2384–2396 (2010).

[b33] Potts B. M. & Pederick L. A. Morphology, phylogeny, origin, distribution and genetic diversity of eucalypts. (CSIRO, 2000).

[b34] Swenson U., Hill R. S. & McLoughlin S. Biogeography of *Nothofagus* supports the sequence of Gondwana break-up. Taxon 50, 1025–1041 (2001).

[b35] Hall R. Cenozoic geological and plate tectonic evolution of SE Asia and the SW Pacific: computer-based reconstructions, model and animations. J. Asian Earth Sci. 20, 353–431 (2002).

[b36] McElhinny M. W. & Embleton B. J. J. Australian palaeomagnetism and the Phanerozoic plate tectonics of eastern Gondwanaland. Tectonophysics 22, 1–29 (1974).

[b37] Schulte J. A., Melville J. & Larson A. Molecular phylogenetic evidence for ancient divergence of lizard taxa on either side of Wallace’s Line. P. Roy. Soc. B-Biol. Sci. 270, 597–603 (2003). 10.1098/rspb.2002.2272 PMC1691285 12769459

[b38] Korall P. & Pryer K. M. Global biogeography of scaly tree ferns (Cyatheaceae): evidence for Gondwanan vicariance and limited transoceanic dispersal. J. Biogeogr. 41, 402–413 (2014). 25435648 10.1111/jbi.12222 PMC4238398

[b39] De Queiroz A. The resurrection of oceanic dispersal in historical biogeography. Trends Ecol. Evol. 20, 68–73 (2005). 16701345 10.1016/j.tree.2004.11.006

[b40] Gladenkov A. Y., Oleinik A. E., Marincovich L. & Barinov K. B. A refined age for the earliest opening of Bering Strait. Paleogeogr. Paleoclimatol. Paleoecol. 183, 321–328 (2002).

[b41] Chen J. J., Cui B. K., Zhou L. W., Korhonen K. & Dai Y. C. Phylogeny, divergence time estimation, and biogeography of the genus *Heterobasidion* (Basidiomycota, Russulales). Fungal Divers. 71, 185–200 (2015).

[b42] Deng T. . Does the arcto-tertiary biogeographic hypothesis explain the disjunct distribution of Northern hemisphere herbaceous plants? The case of *Meehania* (Lamiaceae). PLoS One 10, e0117171 (2015). 25658699 10.1371/journal.pone.0117171 PMC4319762

[b43] Zamora-Tavares M. D. P., Martínez M., Magallón S., Guzmán-Dávalos L. & Vargas-Ponce O. Physalis and physaloids: A recent and complex evolutionary history. Mol. Phylogenet. Eovl. 100, 41–50 (2016). 10.1016/j.ympev.2016.03.032 27063196

[b44] White J. M. . An 18 million year record of vegetation and climate change in north-western Canada and Alaska: tectonic and global climatic correlates. Paleogeogr. Paleoclimatol. Paleoecol. 130, 293–306 (1997).

[b45] Cerling T. E., Harris J. M., MacFadden B. J., Leakey M. G., Quade J., Eisenmann V. & Ehleringer J. R. Global vegetation change through the Miocene/Pliocene boundary. Nature 389, 153–158 (1997).

[b46] Han M. L. . Taxonomy and phylogeny of the brown-rot fungi: *Fomitopsis* and its related genera. Fungal Divers, doi: 10.1007/s13225-016-0364-y (2016).

[b47] Katoh K. & Toh H. Recent developments in the MAFFT multiple sequence alignment program. Brief. Bioinform. 9, 286–298 (2008). 18372315 10.1093/bib/bbn013

[b48] Hall T. A. Bioedit: a user-friendly biological sequence alignment editor and analysis program for Windows 95/98/NT. Nucl. Acids Symp. Ser. 41, 95–98 (1999).

[b49] Gouy M., Guidon S. & Gascuel O. SeaView version 4: a multiplatform graphical user interface for sequence alignment and phylogenetic tree building. Mol. Biol. Evol. 27, 221–224 (2010). 19854763 10.1093/molbev/msp259

[b50] Nylander J. A. A. MrModeltest v2. Program distributed by the author. Evolutionary Biology Centre, Uppsala University (2004).

[b51] Swofford D. L. PAUP*: phylogenetic analysis using parsimony (*and other methods). Version 4.0b10. (Sinauer Associates, 2002).

[b52] Stamatakis A. RAxML-VI-HPC: maximum likelihood-1 based phylogenetic analyses with thousands of taxa and mixed models. Bioinformatics 22, 2688–2690 (2006). 16928733 10.1093/bioinformatics/btl446

[b53] Ronquist F. & Huelsenbeck J. P. Mrbayes 3: Bayesian phylogenetic inference under mixed models. Bioinformatics 19, 1572–1574 (2003). 12912839 10.1093/bioinformatics/btg180

[b54] Lin C. P., Huang J. P., Wu C. S., Hsu C. Y. & Chaw S. M. Comparative chloroplast genomics reveals the evolution of Pinaceae genera and subfamilies. Genome Biol. Evol. 2, 504–517 (2010). 20651328 10.1093/gbe/evq036 PMC2997556

[b55] Eckert A. J. & Hall B. D. Phylogeny, historical biogeography, and patterns of diversification for *Pinus* (Pinaceae): Phylogenetic tests of fossil-based hypotheses. Mol. Phylogenet. Evol. 40, 166–182 (2006). 16621612 10.1016/j.ympev.2006.03.009

[b56] Drummond A. J. & Rambaut A. BEAST: Bayesian evolutionary analysis by sampling trees. BMC Evol. Biol. 7, 214–221 (2007). 17996036 10.1186/1471-2148-7-214 PMC2247476

[b57] Hibbett D. S., Grimaldi D. & Donoghue M. J. Fossil mushrooms from Miocene and Cretaceous ambers and the evolution of homobasidiomycetes. Am. J. Bot. 84, 981–991 (1997). 21708653

[b58] Smith S. Y., Currah R. S. & Stockey R. A. Cretaceous and Eocene poroid hymenophores from Vancouver Island, British Columbia. Mycologia 96, 180–186 (2004). 21148842

[b59] Drummond A. J., Ho S. Y. W., Phillips M. J. & Rambaut A. Relaxed phylogenetics and dating with confidence. PLoS Biol. 4, 88 (2006). 10.1371/journal.pbio.0040088 PMC1395354 16683862

[b60] Clayton J. W., Soltis P. S. & Soltis D. E. Recent long-distance dispersal overshadows ancient biogeographical patterns in a pantropical angiosperm family (Simaroubaceae, Sapindales). Syst. Biol. 58, 395–410 (2009). 20525593 10.1093/sysbio/syp041

