# Supplementary material for: Phylogeny and biogeography of the remarkable genus Bondarzewia (Basidiomycota, Russulales)
Source: Sci Rep. 2016 Sep 29;6:34568. doi: 10.1038/srep34568 (PMC5041112; doi:10.1038/srep34568)
Supplement: Supplementary Information [file srep34568-s1.pdf]

Phylogeny and biogeography of the remarkable genus *Bondarzewia* (Basidiomycota, Russulales)

Jie Song[1+], Jia-Jia Chen[1+], Min Wang[1], Yuan-Yuan Chen[1], Bao-Kai Cui[1]*

Supplementary Legend:

Figure S1 Phylogenetic trees within *Bondarzewia* inferred from the Maximum likehood (ML) analysis based on the ITS, nLSU, mtSSU, and EFA datasets. Branches are labeled for MP/BS and BPP values greater than 50% and 0.95, respectively.

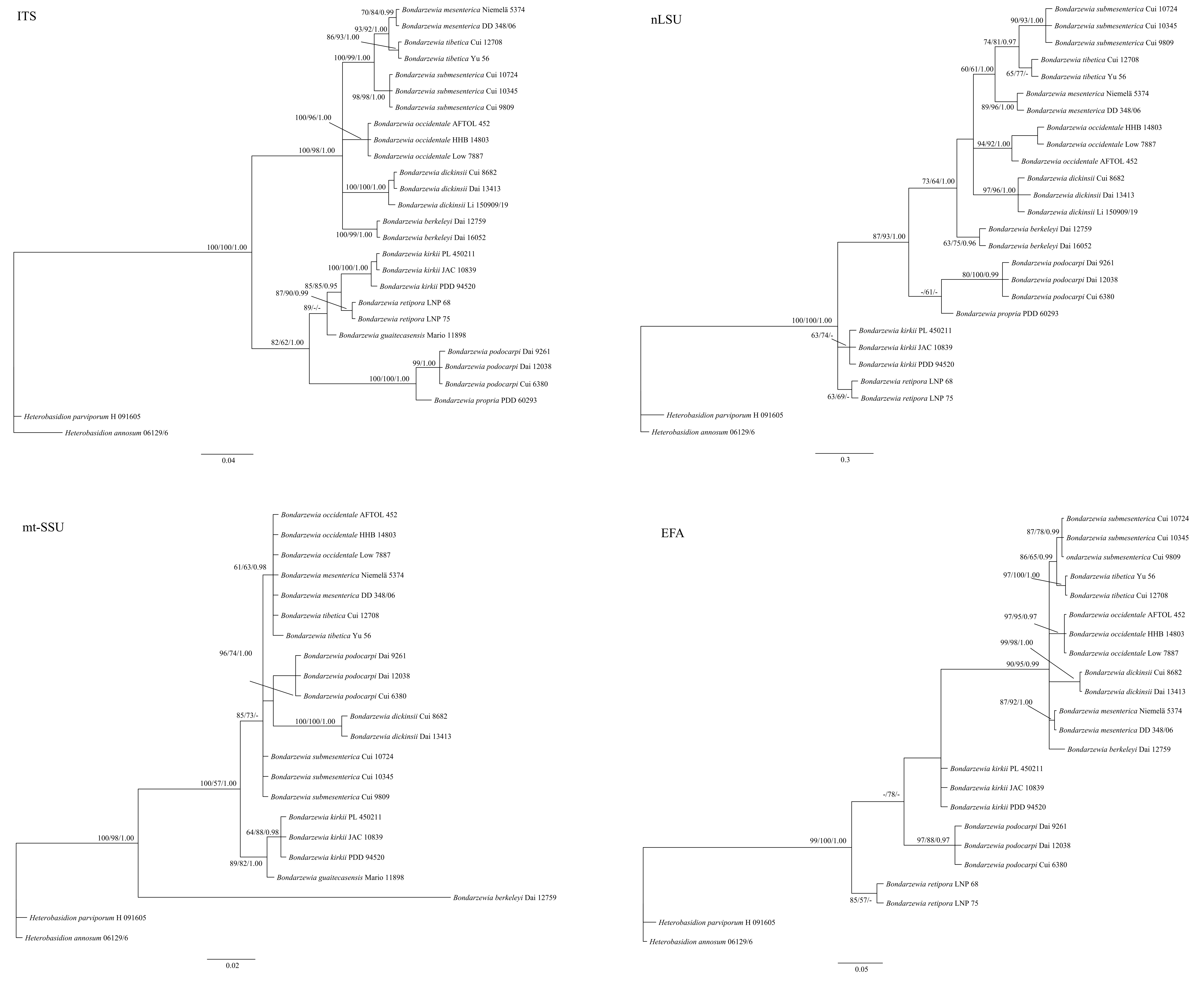

ITS
70/84/0.99
Bondarzewia mesenterica Niemelä 5374
Bondarzewia mesenterica DD 348/06
93/92/1.00
86/93/1.00
Bondarzewia tibetica Cui 12708
Bondarzewia tibetica Yu 56
100/99/1.00
Bondarzewia submesenterica Cui 10724
Bondarzewia submesenterica Cui 10345
98/98/1.00
Bondarzewia submesenterica Cui 9809
100/96/1.00
Bondarzewia occidentale AFTOL 452
Bondarzewia occidentale HHB 14803
100/98/1.00
Bondarzewia occidentale Low 7887
Bondarzewia dickinsii Cui 8682
100/100/1.00
Bondarzewia dickinsii Dai 13413
Bondarzewia dickinsii Li 150909/19
Bondarzewia berkeleyi Dai 12759
100/99/1.00
Bondarzewia berkeleyi Dai 16052
100/100/1.00
Bondarzewia kirkii PL 450211
100/100/1.00
Bondarzewia kirkii JAC 10839
85/85/0.95
Bondarzewia kirkii PDD 94520
87/90/0.99
Bondarzewia retipora LNP 68
89/-/-
Bondarzewia retipora LNP 75
Bondarzewia guaitecasensis Mario 11898
82/62/1.00
99/1.00
Bondarzewia podocarpi Dai 9261
Bondarzewia podocarpi Dai 12038
100/100/1.00
Bondarzewia podocarpi Cui 6380
Bondarzewia propria PDD 60293
Heterobasidion parviporum H 091605
Heterobasidion annosum 06129/6
0.04
nLSU
90/93/1.00
Bondarzewia submesenterica Cui 10724
Bondarzewia submesenterica Cui 10345
Bondarzewia submesenterica Cui 9809
74/81/0.97
Bondarzewia tibetica Cui 12708
60/61/1.00
65/77/-
Bondarzewia tibetica Yu 56
Bondarzewia mesenterica Niemelä 5374
89/96/1.00
Bondarzewia mesenterica DD 348/06
Bondarzewia occidentale HHB 14803
94/92/1.00
Bondarzewia occidentale Low 7887
Bondarzewia occidentale AFTOL 452
73/64/1.00
Bondarzewia dickinsii Cui 8682
97/96/1.00
Bondarzewia dickinsii Dai 13413
Bondarzewia dickinsii Li 150909/19
Bondarzewia berkeleyi Dai 12759
87/93/1.00
63/75/0.96
Bondarzewia berkeleyi Dai 16052
Bondarzewia podocarpi Dai 9261
80/100/0.99
Bondarzewia podocarpi Dai 12038
-/61/-
Bondarzewia podocarpi Cui 6380
Bondarzewia propria PDD 60293
100/100/1.00
Bondarzewia kirkii PL 450211
63/74/-
Bondarzewia kirkii JAC 10839
Bondarzewia kirkii PDD 94520
Bondarzewia retipora LNP 68
63/69/-
Bondarzewia retipora LNP 75
Heterobasidion parviporum H 091605
Heterobasidion annosum 06129/6
0.3
mt-SSU
Bondarzewia occidentale AFTOL 452
Bondarzewia occidentale HHB 14803
Bondarzewia occidentale Low 7887
61/63/0.98
Bondarzewia mesenterica Niemelä 5374
Bondarzewia mesenterica DD 348/06
Bondarzewia tibetica Cui 12708
Bondarzewia tibetica Yu 56
96/74/1.00
Bondarzewia podocarpi Dai 9261
Bondarzewia podocarpi Dai 12038
Bondarzewia podocarpi Cui 6380
85/73/-
100/100/1.00
Bondarzewia dickinsii Cui 8682
Bondarzewia dickinsii Dai 13413
Bondarzewia submesenterica Cui 10724
Bondarzewia submesenterica Cui 10345
100/57/1.00
Bondarzewia submesenterica Cui 9809
Bondarzewia kirkii PL 450211
64/88/0.98
Bondarzewia kirkii JAC 10839
100/98/1.00
Bondarzewia kirkii PDD 94520
89/82/1.00
Bondarzewia guaitecasensis Mario 11898
Bondarzewia berkeleyi Dai 12759
Heterobasidion parviporum H 091605
Heterobasidion annosum 06129/6
0.02
EFA
Bondarzewia submesenterica Cui 10724
87/78/0.99
Bondarzewia submesenterica Cui 10345
86/65/0.99
ondarzewia submesenterica Cui 9809
97/100/1.00
Bondarzewia tibetica Yu 56
Bondarzewia tibetica Cui 12708
Bondarzewia occidentale AFTOL 452
97/95/0.97
Bondarzewia occidentale HHB 14803
99/98/1.00
Bondarzewia occidentale Low 7887
90/95/0.99
Bondarzewia dickinsii Cui 8682
Bondarzewia dickinsii Dai 13413
87/92/1.00
Bondarzewia mesenterica Niemelä 5374
Bondarzewia mesenterica DD 348/06
Bondarzewia berkeleyi Dai 12759
Bondarzewia kirkii PL 450211
-/78/-
Bondarzewia kirkii JAC 10839
Bondarzewia kirkii PDD 94520
99/100/1.00
Bondarzewia podocarpi Dai 9261
97/88/0.97
Bondarzewia podocarpi Dai 12038
Bondarzewia podocarpi Cui 6380
Bondarzewia retipora LNP 68
85/57/-
Bondarzewia retipora LNP 75
Heterobasidion parviporum H 091605
Heterobasidion annosum 06129/6
0.05
